# Novel Protocols for Scalable Production of High Quality Purified Small Extracellular Vesicles from Bovine Milk

**DOI:** 10.7150/ntno.62213

**Published:** 2021-07-05

**Authors:** Spencer R. Marsh, Kevin J. Pridham, Jane Jourdan, Robert G. Gourdie

**Affiliations:** 1 Fralin Biomedical Research Institute at Virginia Tech Carillion, Roanoke, VA, 24016, USA.; 2 Center for Vascular and Heart Research, Virginia Tech, Roanoke, VA, 24016, USA.; 3 Department of Biomedical Engineering and Mechanics, Virginia Tech, Blacksburg, VA, 24061, USA.; 4 Department of Emergency Medicine, Virginia Tech Carilion School of Medicine, Virginia Tech, Roanoke, VA, 24016, USA.; 5 Faculty of Health Science, Virginia Tech, Blacksburg, VA, 24061, USA.

**Keywords:** Exosomes, Small Extracellular Vesicles, Bovine Milk Extracellular Vesicles, Small Extracellular Vesicle Isolation, Tangential Flow Filtration, Drug Delivery vehicle

## Abstract

Extracellular Vesicles (EVs) are cell-secreted nanovesicles that have unique potential for encapsulating and targeting “difficult-to-drug” therapeutic cargos. Milk provides an enriched source of EVs, and of particular interest to the drug delivery field, small EVs. Small EVs are distinguished from large EVs by membrane components, biogenesis mechanism and downstream functionality - in particular, small EVs are primarily composed of exosomes, which show high stability *in vivo* and naturally function in the targeted delivery of biological materials to cells. Moreover, bovine milk is abundantly produced by the dairy industry, widely consumed, and generally well tolerated by humans. Importantly, there is evidence that milk exosomes and small EVs are efficiently taken up into the circulation from the gut, providing the opportunity for their use in administration of therapeutics such as microRNAs or peptides not typically available via an oral route. Unfortunately, present methods for isolation do not efficiently separate EVs from milk proteins, resulting in contamination that is not desirable in a clinical-grade therapeutic. Herein, we present novel EV purification methods focused on optimized timing and levels of temperature and divalent cation chelation. Incorporation of these solubilization steps into centrifugation- and tangential flow filtration-based methods provide large amounts of purified small EVs at ultra-dense concentrations, which are substantially free from contaminating milk proteins. Remarkably, these ultra-dense isolates equal 10 to 15% of the starting volume of milk indicating a prodigious rate of small EV production by mammary glands. Our approach enables gentle, scalable production of ultrastructurally and functionally intact small EVs from milk, providing a path to their industrial scale purification for oral delivery of therapeutic biologics and small drugs.

## Introduction

Exosomes and their overarching classification, Extracellular Vesicles (EVs), are membrane-bound nanovesicles released by cells that act as an evolutionarily conserved mechanism for long-range intercellular signaling 1, 2. In humans and other mammals, EVs are secreted by nearly all cell types and are abundant in most biological fluids including blood, lymph, urine and milk 3, 4. Small EVs (sEVs) are distinct from large EVs, which include apoptotic bodies and microvesicles - structures that are blebbed from the cell membrane during periods of disease 5. Exosomes are categorized within the sEV class, being of a relatively uniform small size, 50-150 nm in diameter, and showing preferential expression of various membrane-associated proteins, including CD81, CD9 and Syntenin, but not others such as Calnexin 6. Small EV constituents can vary, reflecting the type and/or physiological state of the cells from which they were secreted 7. EV cargos include lipids, proteins, and nucleotide sequences (e.g., microRNAs), which can be internally encapsulated or present externally as receptors or adhesion molecules on the vesicular membrane 8, 9. The ability of sEVs to cargo biological signaling molecules *in vivo* has alerted the pharmaceutical industry to their promise as novel and versatile drug delivery devices 10-12. This appeal is further enhanced by the unique ability of certain EVs to cross tissue boundaries such as the cutaneous barrier 13, blood-brain-barrier 14, and gut-blood barrier 15. Small EVs, particularly exosomes, also appear to show varying levels of immune tolerance, with reports that some types of EV are immunologically well-tolerated, even when transferred between individuals and species 16 - further heightening interest in their translational potential as a novel means for improving the delivery and safety of therapeutic molecules.

Presently, one limitation to clinical use of EVs is that a technical approach to their cost-effective purification in large quantities is limited. There are a number of methods for EV isolation, with the current “gold standard” being techniques based on ultracentrifugation (UC). These methods typically involve differential centrifugation steps and/or density gradient UC-based separations. However, the ability to produce EVs in large quantities is restricted by the requirement for multiple UC steps and the fact that UCs can only spin relatively small volumes. It further remains that shearing forces imparted during repeated UC spins may have deleterious effects on EV integrity 17. Other methods that may exert less physical rigor during EV isolation include ultrafiltration, tangential flow filtration (TFF), size exclusion chromatography (SEC), and polyethylene glycol precipitation-based methods. The use of each has been well documented, with the majority of groups utilizing a mix of approaches, as opposed to a single method 18-21.

In recent years, it has been recognized that milk is enriched in EVs, which could offer a source for large-scale production 22, 23. Bovine milk is produced in large quantities by the dairy industry, is widely consumed, and is generally well tolerated by humans. Moreover, milk sEVs have been reported to cross from the gut into the blood circulation and traffic to various organs, including the brain, heart and lungs 24, 25; properties that could provide a basis for oral administration of “drugged” sEVs. Milk comprises a diverse mixture of proteins, minerals, lipids, and other macromolecules- a complexity of composition that poses challenges to EV purification. Casein proteins are a major constituent of milk, making up approximately 80% of all milk proteins. Caseins aggregate into large, colloidal complexes with calcium phosphate and other milk proteins to form what are referred to as Casein micelles. These micelles are approximately 10 nm in diameter and can further coalesce into larger coagulated structures 26. Casein micelle aggregates are thought to bind to and ensnare EVs via hydrostatic interactions, impeding separation from contaminating milk proteins; observations that are confirmed by transmission electron microscopy (TEM) analysis of milk-derived EV preparations 27. Consequently, present methods for isolation of high-purity sEVs from milk are limited by contaminating proteins, such as Casein.

Herein, novel stepwise protocols incorporating chelation of Ca2+ and other divalent cations at specified times and temperatures are described that enable high-yield separation of structurally and functionally intact Small Extracellular Vesicles (sEVs) from milk proteins. These Casein micelle solubilization steps may be incorporated into “gold standard” UC-based approaches for sEV isolation. We also show that our novel approaches can be integrated into more gentle methods of sEV purification, including those incorporating TFF, which to the best of our knowledge has not been reported in the peer-reviewed literature for milk sEV isolation previously. The methods we iterate are the basis of extensive trial and error testing and we provide examples of how, departure from the steps that we describe compromises yield and purity of the final ultra-dense concentrates of sEVs achievable via our optimized approach. The methods described herein may provide a basis for developing industrial scale production of purified, high-quality sEVs as drug delivery devices.

## Materials & Methods

### Small EV Isolation: Ultracentrifuge-Based Method

**Figure 1:**
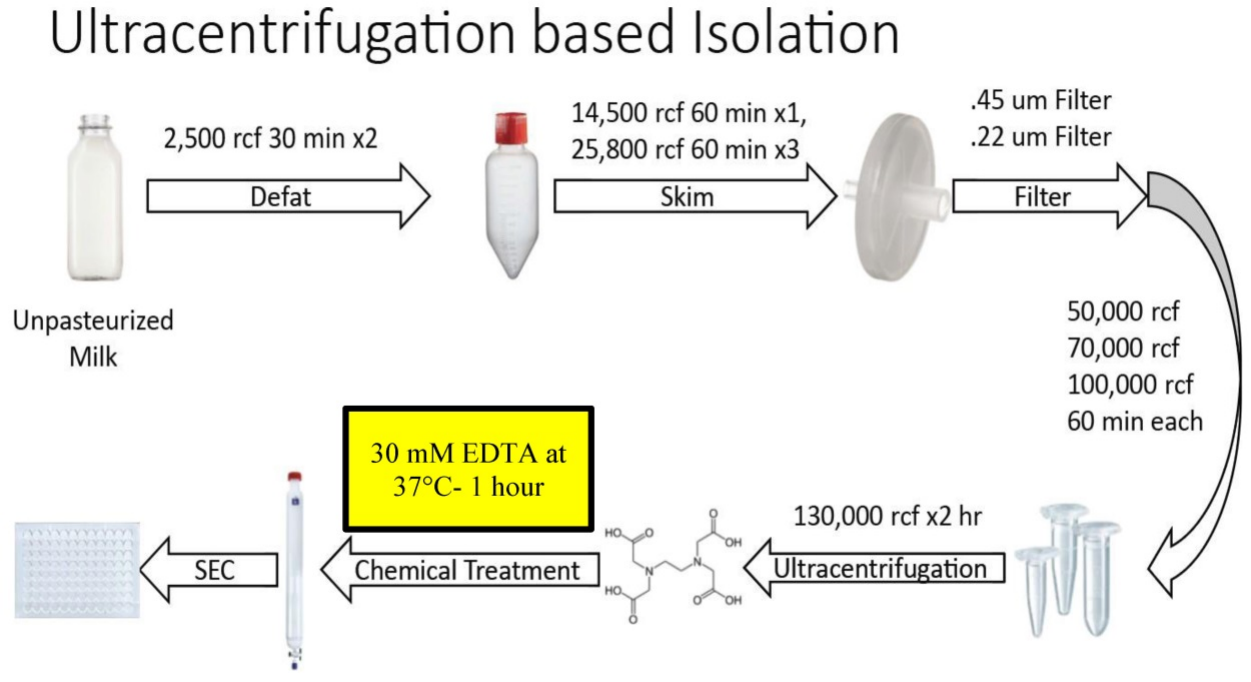
** Overview of steps in the optimized Ultracentrifugation (UC)-based method of small EV isolation from milk.** Chemical chelation with EDTA at 37°C was found to be optimally placed prior to SEC separation.

Figure 1 summarizes the steps of the optimized Ultracentrifugation (UC)-based method. Unpasteurized, full fat, fresh bovine milk at 4°C was obtained from Homestead Creamery of Wirtz, VA after morning milking was completed. All subsequent steps up to the chelation and temperature treatment were performed at 4°C. Milk was transferred to sterile, large polypropylene centrifuge tubes (Thermo Scientific, Waltham MA, 75007585) and centrifuged at 5,000 rcf (Sorval Legend X1R centrifuge with Sorval TX-400 75003629 rotor) for 30 minutes. Fat (cream) was removed either by decanting from the supernatant (SN) or whisking away with filter paper. The remaining SN was transferred to a new container and the pellet discarded. These steps were repeated 2-3 times to ensure defatting. Milk was then transferred to 250 mL centrifugation containers (Nalgene, Rochester NY) and spun at 14,500 rcf (Beckmann Coulter Avanti, Brea, CA; J- 26 XP centrifuge with JLA 16.25 rotor) for 60 minutes. The SN was then transferred to 250 mL polypropylene containers (Beckmann Coulter Avanti) and centrifuged at 22,600 rcf (Beckmann Coulter Avanti J-26 XP centrifuge with JLA 16.25 rotor) for 60 minutes. After each centrifugation, the SN was decanted, the pellet discarded, and any noticeable fat was skimmed. This centrifugation, fat removal and SN decanting step was repeated at 22,600 rcf 3-4 times. The SN was then consecutively filtered through 0.45 µm and 0.22 µm filters (Millipore, Burlington, MA), transferred to Beckmann 355631 ultracentrifuge tubes and spun at 56,000 rcf (Beckmann Coulter Avanti; J-26 XP centrifuge with a JA 25.5 rotor) for 60 minutes. Following these lower speed centrifugations, the pellet was discarded, and the SN transferred to new 355631 Beckmann tubes and spun at 70,000 rcf (Beckmann Coulter Avanti; Optima L-100 XP Ultracentrifuge with SW.32.Ti Rotor) for 60 minutes. Subsequently, SN was transferred to fresh Beckman 355631 tubes, spun at 100,000 rcf (Beckmann Coulter Avanti; Optima L-100 XP Ultracentrifuge with SW.32.Ti Rotor) for 60 minutes. The resulting SN was further centrifuged at 130,000 rcf (Beckmann Coulter Avanti; Optima L-100 XP Ultracentrifuge with SW.32.Ti Rotor) for 120 minutes. The resulting pellet was dissolved (10% by volume) in 2-3 ml of Hepes buffer (100 mM NaCl, 4 mM KCl, 20 mM Hepes, pH 6.7- sterile and degassed) overnight at 4°C. The following morning the solution was triturated and aliquoted at 500 µL. These aliquots were kept at -80°C until further use. Following thawing on ice, EDTA was added to the aliquot at a concentration of 30 mM (or at other concentrations as specified in the results) and the solution was incubated at 37°C for 60 minutes. The solution was then run through an IZON qEV original 70 nm sepharose column (IZON, Christchurch, New Zealand, 1006881) and collected manually in a 96-well plate, with each fraction representing 500 µL of volume. Protein concentrations of resulting fractions were analyzed using a Nanodrop 2000c (Thermo Scientific, ND-2000) running Nanodrop 2000 software on an associated Nanodrop laptop (Thermo Scientific, ND2000LAPTOP) via 260/280 spectrophotometry, using standard methods and Hepes buffer as a blank control solution. After protein quantification, samples were aliquoted and stored at -80°C.

### Small EV Isolation: Tangential Flow Filtration Based Method

**Figure 2:**
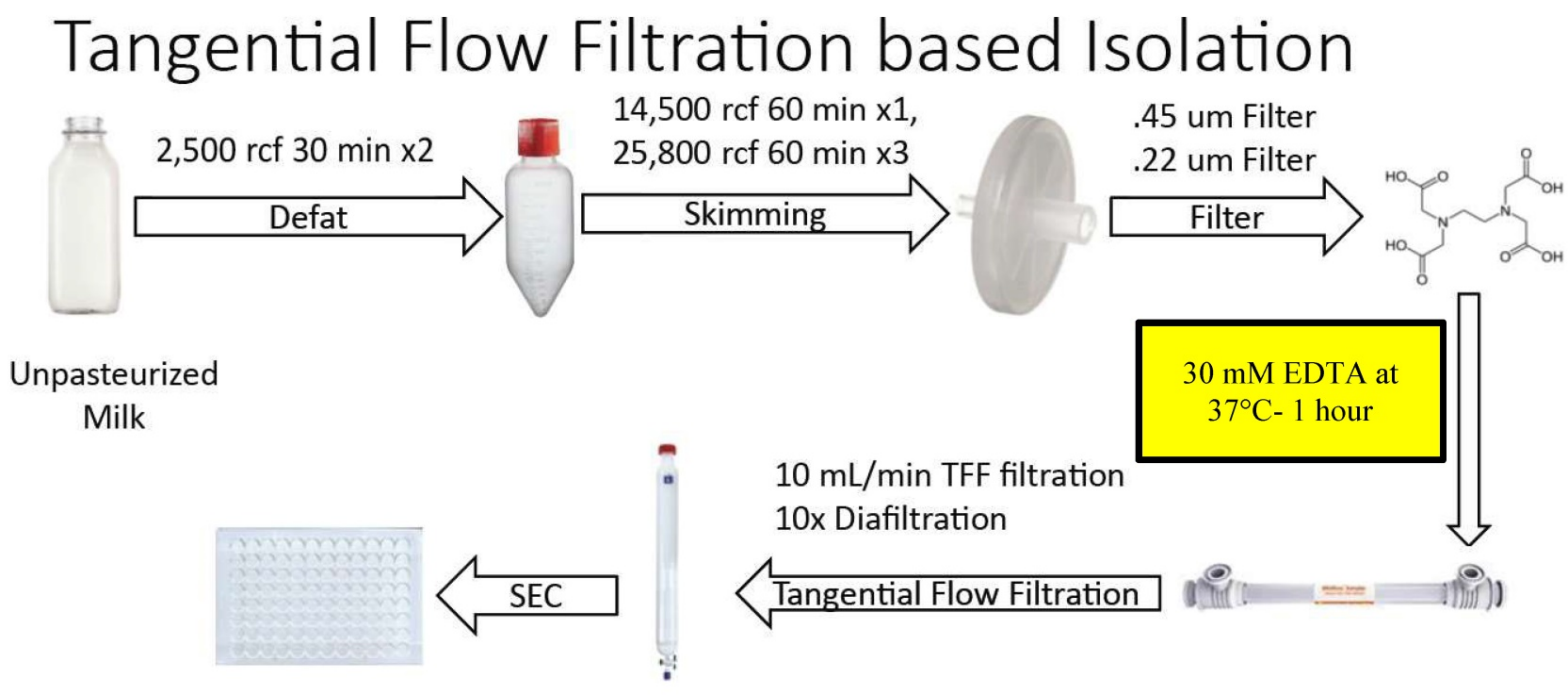
** Overview of steps in the optimized Tangential Flow Filtration (TFF)-based protocol for isolation of small EVs from milk.** Chemical chelation with EDTA 37°C was found to be optimally placed prior to TFF.

Figure 2 summarizes the steps of the optimized Tangential Flow Filtration (TFF)-based method. Unpasteurized bovine milk was obtained at 4°C as per the UC-based method. All subsequent steps up to the chelation and temperature treatment were performed at 4°C. Milk was transferred to sterile large polypropylene centrifuge tubes (Thermo Scientific, 75007585) and processed in identical low speed centrifugation and fat skimming steps as the UC protocol. All other centrifugations up to filtration of the resulting SN by Millipore 0.45 µm and 0.22 µm filters, as performed in the UC-based method, were also performed in this method. The resulting solution was then treated with 30 mM EDTA at 37°C for 60 minutes with gentle stirring. After treatment, the solution was filtered using a Repligen KrosFlo TFF system on a 500 kDa MidiKros TFF Filter (Repligen) at a flow rate of 10 mL/min. Once the filtered solution reached ~10% of the starting volume, the EV-containing solution was further diluted via diafiltration with approximately 10X volume Hepes buffer - composition as for the UC-based method. In turn, once this TFF filtrate reached ~20% of starting volume, the solution was aliquoted and stored in 500 µL volumes at -80°C. Solutions were then separated on an IZON qEV original 70 nm sepharose column (IZON, 1006881), and collected manually in a 96-well plate, with each fraction representing 500 µL of volume. The resulting fractions were analyzed via Nanodrop and spectrophotometry as described in the UC-based method. After protein quantification, samples were aliquoted by fraction and stored at -80°C until subsequent use.

### Gel Electrophoresis and Western Blotting

To prepare for electrophoresis, samples were mixed with Laemelli's sample buffer (Bio-Rad Laboratories, Hercules CA) containing 0.05% beta-mercaptoethanol (Thermo Scientific). Samples were then boiled for 5 minutes at 90°C and 6.25 µg of protein were loaded into each lane of 4%-20% Bio-Rad stain-free gels (Bio-Rad, 5678093). Electrophoresis was performed in standard running buffer (25 mM Tris, 192 mM Glycine, 0.1% SDS) in a Bio-Rad module (Bio-Rad Laboratories, CRITERION Cell 135BR 0030876) for 50 minutes at 200V. The stain free gel was then imaged using a ChemiDoc MP System (Bio-Rad Laboratories) 5-minute activation. Protein transfer from gels was performed in standard transfer buffer (25 mM Tris, 192 mM Glycine, 0.01% SDS) in a Bio-Rad Trans-blot Turbo at 25V and 1.0A for 30 minutes onto a PVDF (MilliporeSigma, St. Louis MO, IPFL00010) membrane. Subsequently, the membrane was dried at RT for 1 hour to affix proteins. The PVDF transfer membrane was then rehydrated in methanol, washed in distilled water and blocked in 3% Fish Skin-Gelatin Extract (FSE) (Thermo Scientific) in TBST (20 mM Tris, 150 mM NaCl, 0.1% Tween-20, pH 7.6) for 1 hour at room temperature. Overnight primary antibody incubation was performed as directed by manufacturer instructions. Antibodies were diluted in 3% FSE in TBST and left overnight at 4°C on a horizontal rocker. Antibodies were diluted as follows: CD81 (Cell Signaling Technology, Danvers MA, 56039S, 1:1,000), CD9 (Novus Biologicals, Littleton CO, NB500-494, 1:1,000), Calnexin (MilliporeSigma, AB2301, 1:1,000), Casein (Abcam, Cambridge UK, ab166596, 1:2,000), ARF6 (Novus Biologicals, NBP1-58310, 1:1,000), Syntenin-1 (Santa Cruz Biotechnology, Dallas TX, SC-100336, 1:1,000). The membrane was then washed 5x in TBST for 5 minutes at RT on an orbital shaker (VWR, Radnor PA, 100 10M0219G) to remove non-bound antibody. Following washing, the membrane was incubated for 1 hour at RT in secondary antibodies diluted 1:20,000 for mouse (Jackson ImmunoResearch, West Grove PA, 715-035- 150) and 1:30,000 for rabbit (Southern Biotechnology, Birmingham AL, 4050-05) in 1:1 TBST:3% FSE, then was washed 5x in TBST for 5 minutes on a shaker. The blot membrane was then incubated in Thermo Scientific Pico activation buffer for 5 minutes and imaged on a Bio-Rad ChemiDoc MP imager under Chemi-detection settings.

### Nanoparticle Tracking Analysis

Nanoparticle Tracking Analysis (NTA) was performed on a NanoSight NS300 (Malvern Panalytical, Malvern, UK) at RT. EV concentrates obtained post-SEC were diluted 1:10 in Hepes buffer, then underwent bath sonication in a Branson 2510 bath sonicator (Branson, Danbury CT) for 30 seconds at RT to reduce sample aggregation. EVs were then diluted (1:1,000 to 1:10,000 depending on sample) and added to a 1 mL syringe, then set on a syringe pump (Malvern Panalytical) and loaded into the NanoSight low volume flow cell. Each sample was analyzed using a 405 nm laser with 5 consecutive 1 minute video recordings with a constant flow rate set at 10 (no units), flow rates are set in the software and do not contain units. Videos were compiled and analyzed in the NTA software (Version 3.4). All videos were compiled and analyzed together in the NTA software and data were collected and saved in raw form.

### Laser Scanning Confocal Microscopy of Calcein Uptake

Small EV concentrates post-SE were diluted 1:10 in Hepes buffer then incubated at 37°C with Calcein-AM (Thermo Scientific, C1430) at 10 uM at 1, 2, 3 or 4-hour intervals. After incubation, extravesicular dye was removed with Sepharose G50 spin columns (USA Scientific, Ocala FL 1415-1601) pre-equilibrated with Hepes buffer. 6 µL of this solution was then dispensed onto a microscope slide (Premiere Scientific, Grand Prairie TX, 75x25x1 mm, 9105) and cover-slipped (Thermo Scientific, 12541A). Calcein intensity and dye retention in particles suspended in this solution were monitored directly by optical sectioning using a 63x objective lens (oil, 1.4 NA) on a Leica SP8 confocal microscope (Leica Camera AG, Wetzlar Germany) with 488 laser, HyD, 1AU, and scan frequency of 700Hz for 6 fields per slide.

### Transmission Electron Microscopy

Formvar-coated 200 mesh copper grids (Electron microscopy sciences, Hatfield PA, FCF200-CU) were glow discharged on a Pelco glow discharge unit (Pelco, Fresno CA) at 0.29 mBAR for 1 minute. 0.1% poly-L-Lysine was applied to the grid for 1 minute and excess solution wicked away with Whatman (Whatman PLC, Maidstone UK) #1 filter paper. Grids were washed 2x with 10 µL milli-Q (Millipore Sigma) water and excess liquid was removed with filter paper. Grids were then dried overnight at RT. Samples were loaded by applying 10 µL of prepared SEC sEV concentrate to the grid for 5 minutes. Excess solution was wicked off with filter paper, the sample was then negatively stained with 10 µL Uranyless stain (Electron microscopy sciences, 22409) for 1 minute at RT. Excess stain was then wicked off. The grid was left to dry overnight at RT before transmission electron microscope (TEM) imaging. Imaging of negatively stained preparations was performed on a FEI Tecnai G20 Biotwin TEM (FEI Company, Hillsboro OR) at 120 kV and images were captured using an Eagle (GATAN, Pleasanton, CA) 4K HS camera.

## Results

### Optimized Ultracentrifugation-Based Isolation Protocol

**Figure 3:**
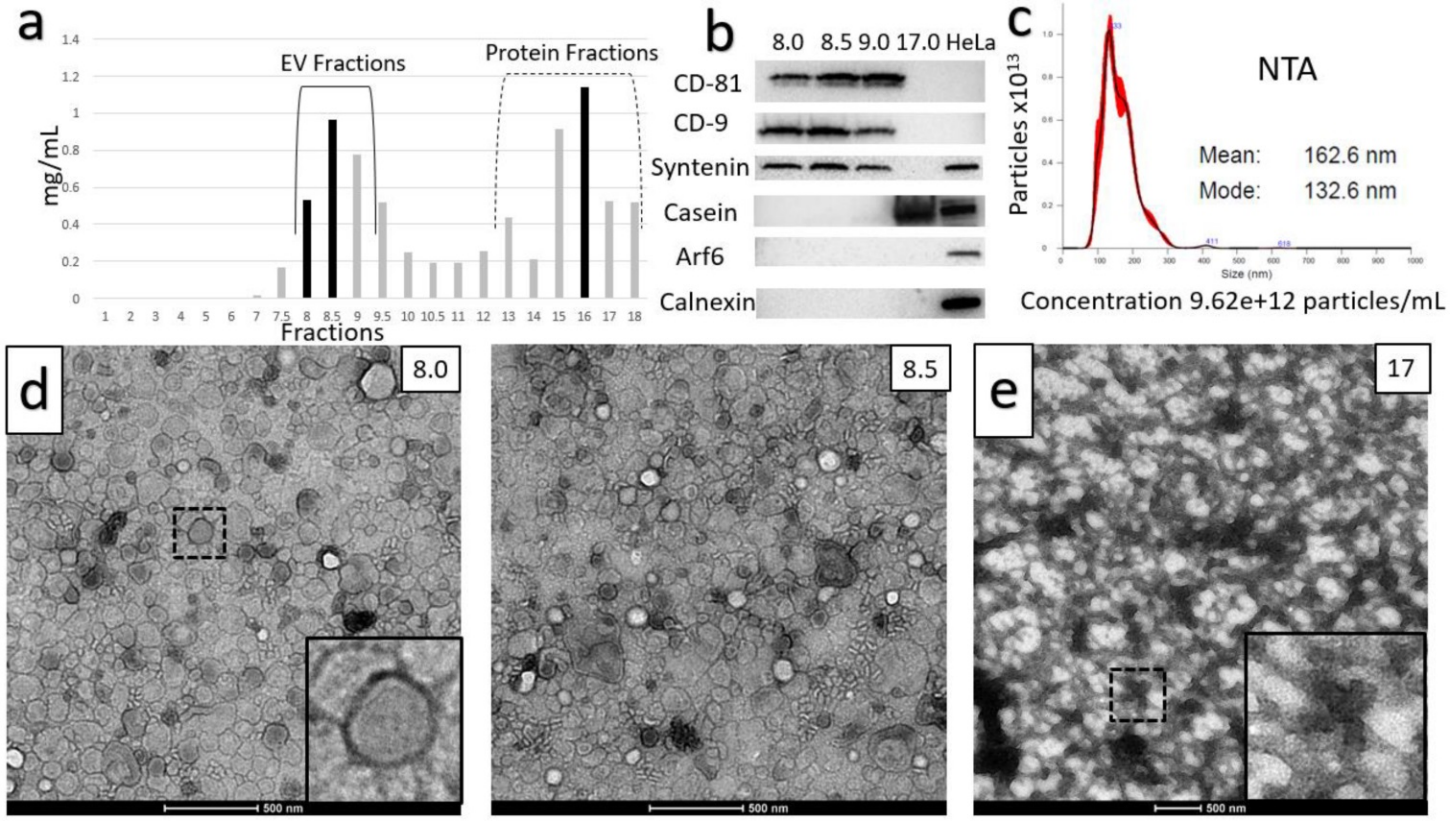
** Characterization of the Ultracentrifuge-based method for small EV (sEV) isolation.** A) Sequential fractions collected during the SEC filtration step, with protein concentrations in mg/ml. B) Western blot of sEV markers CD-81, CD-9 and Syntenin, along with non-small EV markers: Casein, Arf6 (microvesicle marker) and calnexin (endoplasmic reticulum and apoptotic body marker). Peak sEV SEC fractions occur between fractions 8 and 9. Contaminating proteins, including Casein, predominate after fraction 12. Lysates from HeLa cells are included as comparative controls. C) Nanoparticle Tracking Analysis (NTA) data for sEV isolates. Concentration is shown under NTA graph. D) Negative stain electron microscopy of final isolates, showing ultra-dense accumulation of sEVs in peak SEC fractions, and E) high levels of Casein macrostructures in a later SEC isolate (fraction 17).

Two distinct protocols were optimized for isolation of purified small EVs (sEVs) from milk. The key step in each of the protocols was chemical solubilization of Casein micellar structures by divalent cation chelation with 30 mM EDTA at 37°C for 1 hour. The first protocol is referred to as the Ultracentrifugation (UC)-based method (Figure 1). The second incorporated Tangential Flow Filtration (TFF) and is referred to subsequently as the TFF-based protocol or method (Figure 2). In the UC-based method, high levels of EV yield and purity were achieved by placement of the primary chelation step prior to the final Sepharose Column (SEC) filtration step (Figures 1 and 3). The histogram in Figure 3A shows sequential fractions collected during SEC filtration, with protein concentrations measured by nanodrop in mg/ml. Western blotting in Figure 3B demonstrates signals for the sEV markers CD81, CD9, and Syntenin, in tandem with the absence of bands corresponding to Calnexin (an endoplasmic reticulum/cell marker) 28 and Arf6 (microvesicle/large EV marker) 29. Casein is undetectable in the peak sEV SEC fractions 8.0, 8.5 and 9.0 (Figure 3B). HeLa whole cell lysate served as a control. Small EV protein markers were absent from later SEC fractions 15-18 (e.g., Figure 3B). Nanoparticle Tracking Analysis (NTA) was performed and the size distribution and concentration of sEVs were calculated (Figure 3C) on the peak SEC fraction 8.5. The NTA analysis indicated particle sizes consistent with sEVs (mode ~ 133 nm) at a concentration of ~9.6x10^12^ particles/mL. The TEM images in Figure 3D show negative staining of densely packed sEVs in peak SEC fractions (8.5 and 9). Figure 3E shows Casein macrostructures in fraction 17 - a typical Casein micelle found in these fractions is shown at higher magnification in the inset. In sum, TEM, NTA and Western blotting confirm the presence of relatively pure and ultra-structurally definitive sEVs at very high density in peak SEC fractions 8.0 through 9.0, with low levels of protein signal and particulate matter corresponding to Casein and Casein micellar aggregates. Notably, from a typical starting amount of 1000 mL of milk at the beginning of the UC-based protocol, these final ultra-dense sEV concentrates comprise an average of 75 ml (+/-10 mL) - i.e., 7.5% of starting volume (Figure 3).

Shearing forces imparted during UC are thought to have deleterious effects on EV structure 30, 31. Our observations also suggest that UC may have effects on the yield and purity of sEVs from milk. The composition of pellets from the 70,000 RCF and 100,000 RCF spins, along with supernatant, concentrated via TFF, after the 130,000 RCF spin of the UC-based method, are shown in Supplemental Figures 1 and 2, respectively. SEC filtration of these samples followed by TEM indicated the presence of large numbers of sEVs in all of these samples. As these pellets and supernatant are discarded, the large numbers of EVs present in these fractions would be lost; reducing the potential yield from the optimized UC-based method.

### Optimized TFF-Based Isolation Protocol

**Figure 4:**
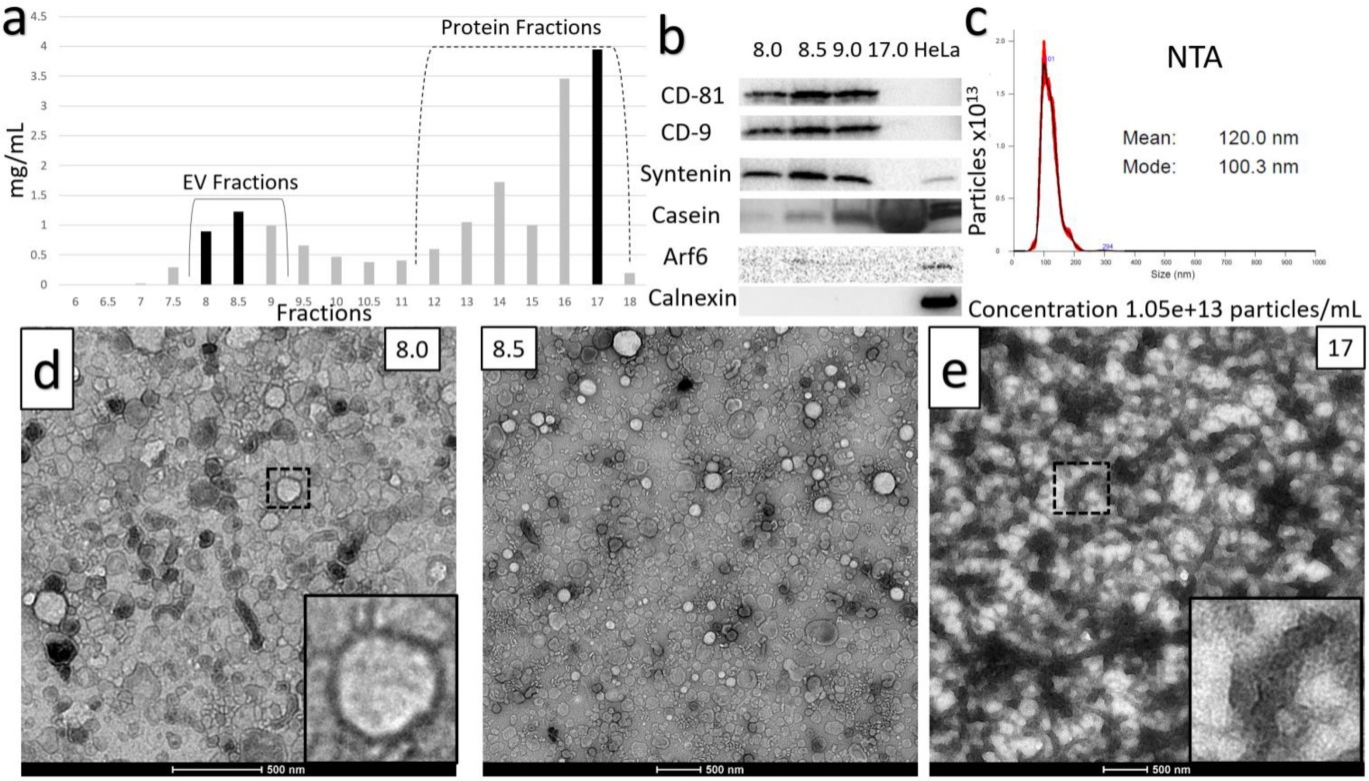
** Characterization of TFF-based protocol for small EV isolation.** A) Sequential fractions collected during the SEC filtration step, with protein concentrations in mg/ml. B) Western blot of sEV markers CD-81, CD-9 and Syntenin, along with non-small EV markers Casein, Arf6 and calnexin. Peak sEV SEC fractions occur between fractions 8 and 9. Contaminating proteins, including Casein, predominate after fraction 12. Lysates from HeLa cells are included as comparative controls. C) Nanoparticle Tracking Analysis data for sEV isolates. Concentration is shown under NTA analysis graph. D) Negative stain electron microscopy of final isolates, showing ultra-dense accumulation of sEVs in peak SEC fractions 8.5 and 9, and E) high levels of Casein macrostructures in a later SEC isolate (fraction 17).

**Figure 5:**
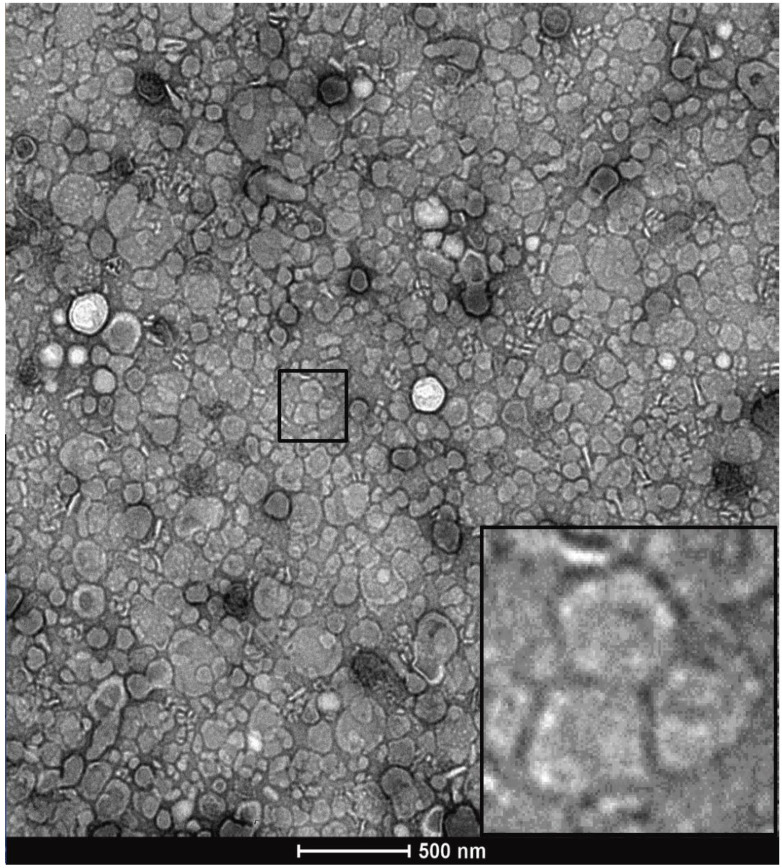
Representative TEM image of post-SEC, EV-containing fraction number 8.5.

The loss of sEVs illustrated in Supplemental Figures 1 and 2 during the UC-based method led us to explore TFF as an alternative. The optimized protocol for TFF-based isolation of sEVs developed from our studies is summarized in Figure 2. The histogram acquired from protein concentrations of sequential SEC fractions following TFF is shown in Figure 4A, with protein concentrations in mg/mL. Findings from the TFF-based protocol paralleled the UC-based method in many respects. However, a key difference was that the 30 mM EDTA/37°C Casein solubilization step was found to be optimally placed before TFF, rather than prior to SEC filtration. Additionally, SEC was found to be required for optimized isolation of sEVs - ensuring separation from contaminating proteins and large EVs. Whilst TFF proved adept at prodigiously concentrating the EV solution, repeated diafiltration rounds in TFF were found to result in diminishing returns, i.e. experiments performed with multiple diafiltrations were comparable to our proposed protocol, which used 10X volume diafiltration. With SEC incorporated, final yields for the TFF-based protocol were ~ 100% higher than the UC-based method. Western blots in Figure 4B are from peak sEV SEC fractions 8 through 9 and show the presence of high levels of the sEV markers CD81, CD9 and Syntenin, along with the absence of Calnexin and Arf6, in these fractions. Casein is extremely reduced in peak SEC fractions containing EVs produced by the TFF-based method relative to the heavy expression found in the late fractions, i.e. Fraction 17 (Figure 4B). The NTA analysis shown is from the peak fraction 8.5 and indicates a mode of 100 nm at a concentration of over 1x10^13^ particles/mL in the final solution (Figure 4C). The TEM images below the histogram (Figure 4D) show ultra-dense, pure sEV concentrates in the peak SEC fractions (8.5 and 9.0), while TEM of a later SEC fraction (17) indicate high levels of Casein micelle aggregates (Figure 4E). The TFF-based protocol provided an average of 125 ml (+/- 20 mL) of EV concentrate in its peak fractions per 1,000 mL of milk, i.e., ~12.5% of the starting volume of milk (Figure 5).

**Figure 6:**
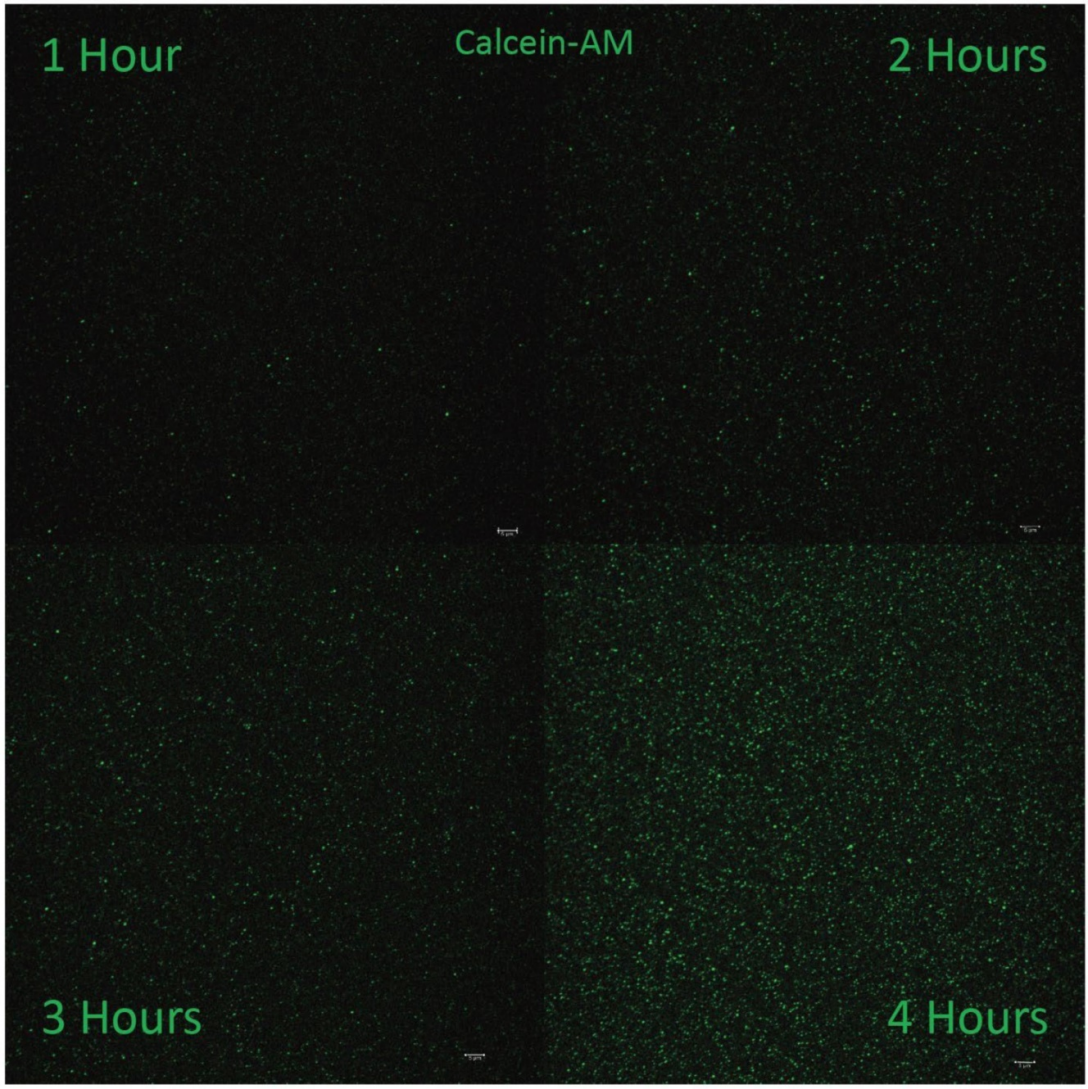
** Time-dependent uptake of esterified Calcein AM dye into TFF-isolated milk EVs**. Peak small EV containing SEC fractions were diluted 1:10 in Hepes buffer. The images show uptake resulting from 1-, 2-, 3- and 4-hour incubations in Calcein-AM. Dye uptake indicate that the sEVs contain esterase activity and are capable of retaining increasing amounts of de-esterified calcein molecules over at least a 4-hour time course. Scale bars in bottom right of each image represent 1 µm.

Figure 6 illustrates confocal optical sections of Calcein-labeled sEVs in peak SEC fractions suspended 1:10 in Hepes buffer generated by the TFF-based method. The images illustrate uptake resulting from 1-, 2-, 3- and 4-hour incubations in Calcein-AM - a dye that is non-fluorescent until activation by de-esterification. The punctate fluorescent signal suggests that the isolated EVs contain esterase activity and are capable of retaining de-esterified Calcein molecules. The level of Calcein signal becomes more intense with longer incubation, suggestive of the cumulative retention of dye and the structural/functional integrity of the isolated EVs. Similar patterns of Calcein fluorescence and retention were observed in sEVs isolated using the UC-based protocol.

### Effects of Deviation from Optimized Protocols

For the purpose of comparison, supplemental figure 3A shows the typical TEM negative-stain appearance of sEVs isolated using the UC-based method, but without the final Casein solubilization and SEC filtration steps, as implemented in the optimized protocol. Small EVs are ultra-structurally evident, though at significantly lower density than in the optimized protocols (compare to Figures 3 and 4). There is also an abundance of Casein micelles accompanying the sEVs, which are not present when optimized methods are used. Supplemental figures 3B-3D illustrate examples of other sub-optimal outcomes, in terms of sEV density and Casein contamination: For example, when the SEC filtration (Supplemental Figure 3B), 37°C temperature (Supplemental Figure 3C) or divalent cation chelation (Supplemental Figure 3D) aspects of our optimized protocols are omitted. In experiments where divalent cation chelation or 37°C temperature incubation were not carried out, sEV densities were decreased and Casein contamination increased in peak SEC fractions, as evidenced by the ultrastructural presence of Casein micelles and 30 to 35 kDa gel bands corresponding to Casein in these same SEC fractions. When the peak fractions from these experiments were immunoblotted for CD-81, reductions in band intensities were observed, consistent with the lower yields and increased proteinaceous contamination observed by TEM (Supplemental Figures 3C-3F). We also investigated the effects of Casein solubilization at varying EDTA concentrations, times, and temperatures. These deviations from the optimized protocol further highlighted the specificity and necessity of the EDTA solubilization steps we describe in the optimized methods. Specifically, use of EDTA at concentrations less than 30 mM (Supplemental Figure 3E) or for less than 1 hour (Supplemental Figure 3F), resulted in increased Casein contamination and reduced EV density, as evidenced by TEM and gel-based analyses. All such deviations culminated in peak SEC fractions exhibiting lower sEV densities, coupled with increased levels of Casein contamination, similar to the results shown in supplemental figures 3A-3D. Incubations in 30 mM EDTA for up to 2 hours, though not deleterious, appeared to provide no further benefit in terms of sEV yield or solubilization of contaminating milk proteins, whereas EDTA concentrations above 30 mM caused bleb-like deformations to sEV ultrastructure - indicative of loss of membrane integrity (Supplemental Figure 4). We also investigated the effect of reducing the number of ultracentrifugation runs, by skipping directly to the 130,000 rcf spin after 0.45 and 0.22 um filtration and determined that this increased levels of contaminating Casein in the peak fraction following SEC relative to optimized methods (Supplemental Figure 5).

## Discussion

Variation in the purity of small EVs (sEVs) produced by isolation protocols, including the differential presence of extracellular vesicular subtypes and contamination by proteinaceous aggregates is an issue that impedes progress in the field 32. The problem of contamination is a particular concern when isolating sEVs from milk, where Casein micelles and higher order polymeric structures containing Casein, routinely co-sediment during purification of EV fractions 33. In the present study, we provide approaches to significantly reduce the burden of contaminant proteins in sEV isolates from milk. Central to our methods is the strategic deployment of a divalent cation chelation treatment at 37°C that promotes solubilization of Casein micelles. When this one-hour treatment is used at specific junctures of the TFF- and UC - based methods described herein, efficient separation of sEVs from Casein-containing aggregates can be achieved. Deviation from optimized protocols, including use of concentrations of EDTA less than or more than 30 mM and incubation at temperatures below 37°C, as well as deployment of the chelation step at stages within our methods other than those that are detailed in Figures 1 and 2, result in isolates of lower purity, higher levels of contamination by milk proteins and/or degradation of EV ultrastructure. Attempts to eliminate SEC separation from the procedure resulted in significantly reduced concentrations and sample purity, highlighting the necessity of each step in the protocol. Additionally, the inherent complexity of milk reinforces the need for each step, as evidenced by the high levels of Casein present in late SEC fractions (i.e. Fraction 17), further supporting the use of each procedural step. Whilst theory suggests TFF repetition might serve to replace the final SEC separation step in the methods we describe herein, in practice it was determined that TFF exhibited diminishing returns when diafiltration was repeated multiple times. Based on the trial and error approach taken, we concluded that a terminal SEC separation step was required for optimal yield, concentration and purity of EVs generated by our methods.

A further impediment to research progress in the field is the current limited ability to produce sEVs cheaply and efficiently at large scale. Large starting volumes of body fluids or tissue (e.g., plasma, urine, adipose tissue), or cell-culture media are typically required, and even then, yields of final isolates tend to be modest 31. Our methods enable large volumes of purified sEVs to be produced at high density from relatively modest starting volumes of milk in a cost-effective, straightforward series of steps. Indeed, the extent to which sEVs make up a significant fraction of milk by volume (10-15 % for the TFF optimized protocol) was an unexpected result from our study. The fact that milk is packed to this degree with EVs, many of which have been reported in the literature to contain miRNAs and other molecules with informational or signaling potential 34, places the developmentally instructive versus nutritional functions of mammalian nursing in an interesting new light.

The difference in absolute yield obtained from the TFF- and UC-based methods is notable. The ultra- dense accumulations of sEVs in peak SEC fractions resulting from the TFF-based method (e.g., Figure 5), are equivalent to ~12.5% of the starting volume of milk. The UC-based method yields EV concentrates at a still impressive ~7.5% of starting volume (Figure 3). Our TEM analyses suggest that the lower yield of the UC-based method may be due to the lower efficiency of this protocol, as illustrated in supplemental figures 1 and 2. In light of the yield of sEVs obtained by the TFF-based method, it appears to be the preferred approach. This preference is reinforced by Western blotting and NTA results indicating that sEVs generated by the two methods are relatively comparable in terms of purity and particulate densities per unit volume. A further consideration is that protocols incorporating continuous-flow TFF separation may be more inherently scalable than those reliant on multiple UC steps - potentially giving a basis for industrial scale production of sEVs from milk.

The large amounts of pure sEVs generated by our methods provide an ample basis for ongoing experimentation and method testing, including the development of technical approaches to loading sEVs with cargoes such as small drugs and peptides, large macromolecular drugs, and miRNAs. Safe and efficacious drug delivery in animal models has been shown for drugs cargoed by exosomes and sEVs including doxorubicin 14, 35, 36, curcumin 37, and paclitaxel 38, as well as siRNAs 39 and miRNAs 40, 41. Techniques for loading exogenous molecules into EVs reported in the literature include electroporation, sonication, freeze-thawing, extrusion and membrane saponification 41, 42. Whilst somewhat effective, a drawback of such techniques is damage to EV membranes - decreasing drug retention and effective delivery of therapeutic cargos to cells. Results from TEM analyses on sEV fractions from various iterations of our isolation protocols suggest that these membranes can be sensitive to mechanical and chemical disruption (e.g., Supplemental Figures 3 & 4). Moreover, a novel and relatively gentle approach to drug loading is suggested by our Calcein retention assay in Figure 6, wherein uptake and retention of exogenous molecules into sEVs might be enhanced by the addition of ester groups to cargo molecules prior to loading. Our preliminary data with short therapeutic peptides based on the Connexin 43 (Cx43) carboxyl terminus 43, suggest that esterification is worthy of further investigation as a strategy for EV drug loading.

In conclusion, we describe new a methods for producing large amounts of pure small EVs at ultra-dense concentrations from milk. This abundance of material to work with should improve the feasibility of tests on optimized storage conditions, cargo and biomarker characterization, vesicular surface functionalization, and drug loading, as well as studies *in vivo* of biodistribution and safety and efficacy of EV-based therapies. Most importantly, the TFF-based technique that we describe may provide a basis for methods of large-scale production of sEVs for the pharmaceutical industry as drug delivery devices.

## Supplementary Material

Supplementary figures.

## Acknowledgements and Funding Support

The authors would like to offer effusive thanks to Homestead Creamery, Inc. of Wirtz, VA for their generosity and support of our work. Particularly we would like to thank Mr. Donnie Montgomery, President of Homestead Creamery for providing ready access to high quality raw milk for our studies. This work was supported by the U.S. National Institutes of Health R01 grants 2R01HL056728-18 and 5R01HL141855-03, and a grant from the Commonwealth Research Commercialization Fund of the Virginia Center for Innovative Technology (CIT) to R.G. Gourdie. The authors would also like to thank and acknowledge Dr. Joy Wolfram (Mayo Clinic, Jacksonville, FL) for advice and information regarding the use of TFF in EV purification.

### Author Contributions

Conception and Design: R.G.G. and J.J. conceived the project. S.R.M., J.J., and K.J.P. designed the project. Development of Methodology: J.J., S.R.M., K.J.P., and R.G.G. Acquisition of Data: K.J.P., S.R.M., and J.J. performed the experiments. Analysis and Interpretation of Data: S.R.M., K.J.P., J.J, and R.G.G. Writing, Review, and/or Revision of the Manuscript: S.R.M., R.G.G., K.J.P., and J.J. All authors reviewed the manuscript.

## Abbreviations

EV: Extracellular Vesicle
sEV: Small Extracellular Vesicle
UC: Ultracentrifugation
TFF: Tangential Flow Filtration
SEC: Size Exclusion Chromatography
TEM: Transmission Electron Microscopy
SN: Supernatant
EDTA: Ethylenediamenetetraacetic Acid
WB: Western Blot
FSE: Fish Skin Gelatin Extract
PVDF: Polyvinylidene difluoride
NTA: Nanoparticle Tracking Analysis
RT: Room Temperature
siRNA: Small Interfering RNA
miRNA: Micro-RNA
Cx43: Connexin-43
