# Supplementary material for: Novel Protocols for Scalable Production of High Quality Purified Small Extracellular Vesicles from Bovine Milk
Source: Nanotheranostics. 2021 Jul 5;5(4):488–98. doi: 10.7150/ntno.62213 (PMC8342262; doi:10.7150/ntno.62213)
Supplement: Supplementary file 1 — Supplementary figures. [file ntnov05p0488s1.pdf]

Supplemental Figures:

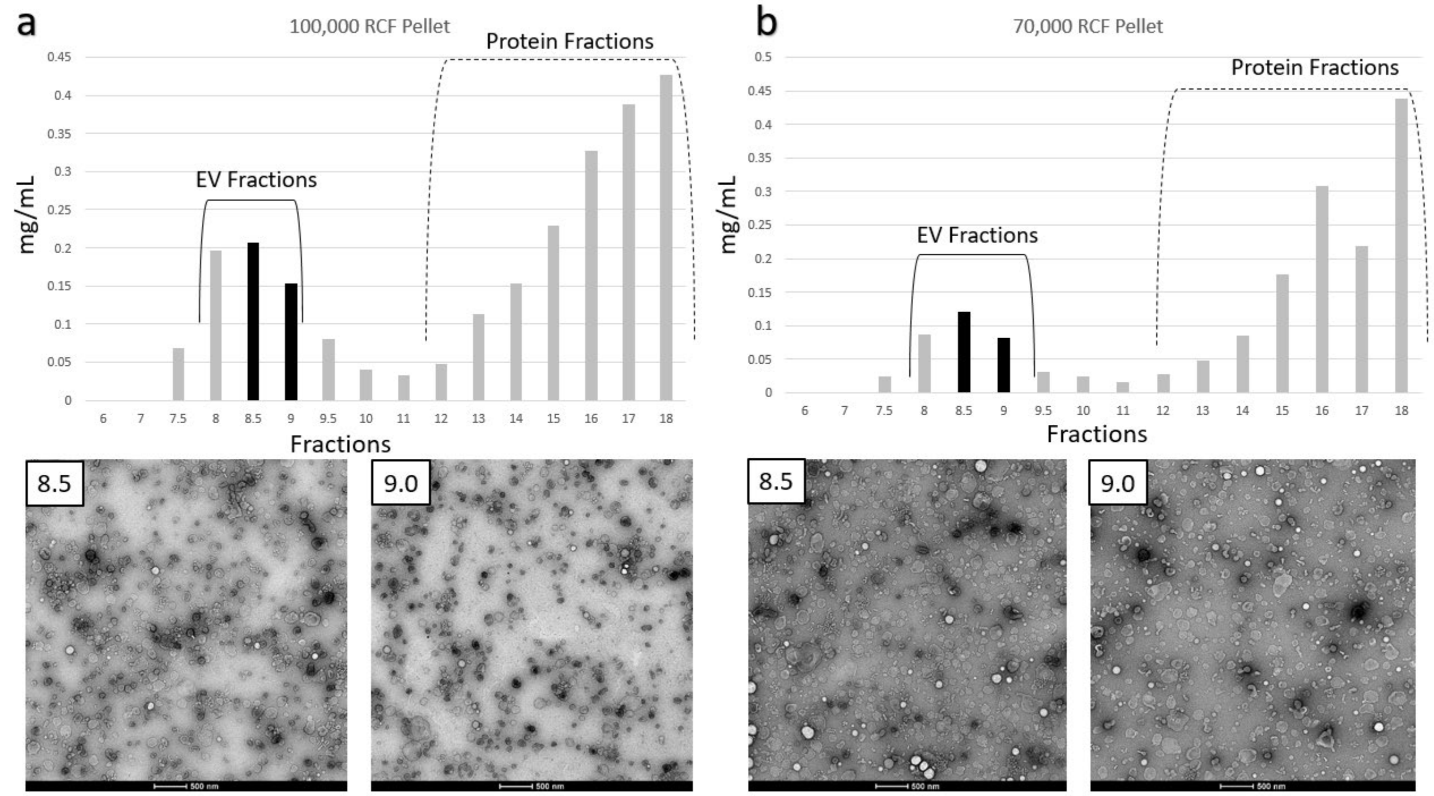

Supplemental Figure 1: Characterization of potential sources of small EV loss during the UC-based method. A) Composition of the 100,000 RCF spin pellet as indicated by SEC filtration, with protein concentrations of the sequential fractions given in mg/ml. Shown below the SEC fractionation chart are negative stain electron microscopy images of fractions 8.5 and 9.0, which typically contain the peak density of EVs. Following the 100,000 RCF spin the supernatant is collected and then subject to pelleting in a final 130,000 RCF ultracentrifugation, with subsequent solubilization in the EDTA step being undertaken on the pelleted material as in Figure 1. The EVs pelleted by the 100,000 RCF spin, as shown in the figure, are not harvested and are instead lost to this final processing step, reducing the yield EVs. **B)** Composition of the 70,000 RCF pellet as indicated by SEC filtration, with protein concentrations in mg/ml. Shown below the chart are negative stain electron microscopy images of fractions 8.5 and 9.0. The 70,000 RCF spin is the centrifugation step prior to the 100,000 RCF step that is characterized in panel (A) of this figure. Significant numbers of EVs can also be pelleted from the 70,000 RCF spin, again indicating a source of inefficiency in the UC-based method in harvesting all available EVs.

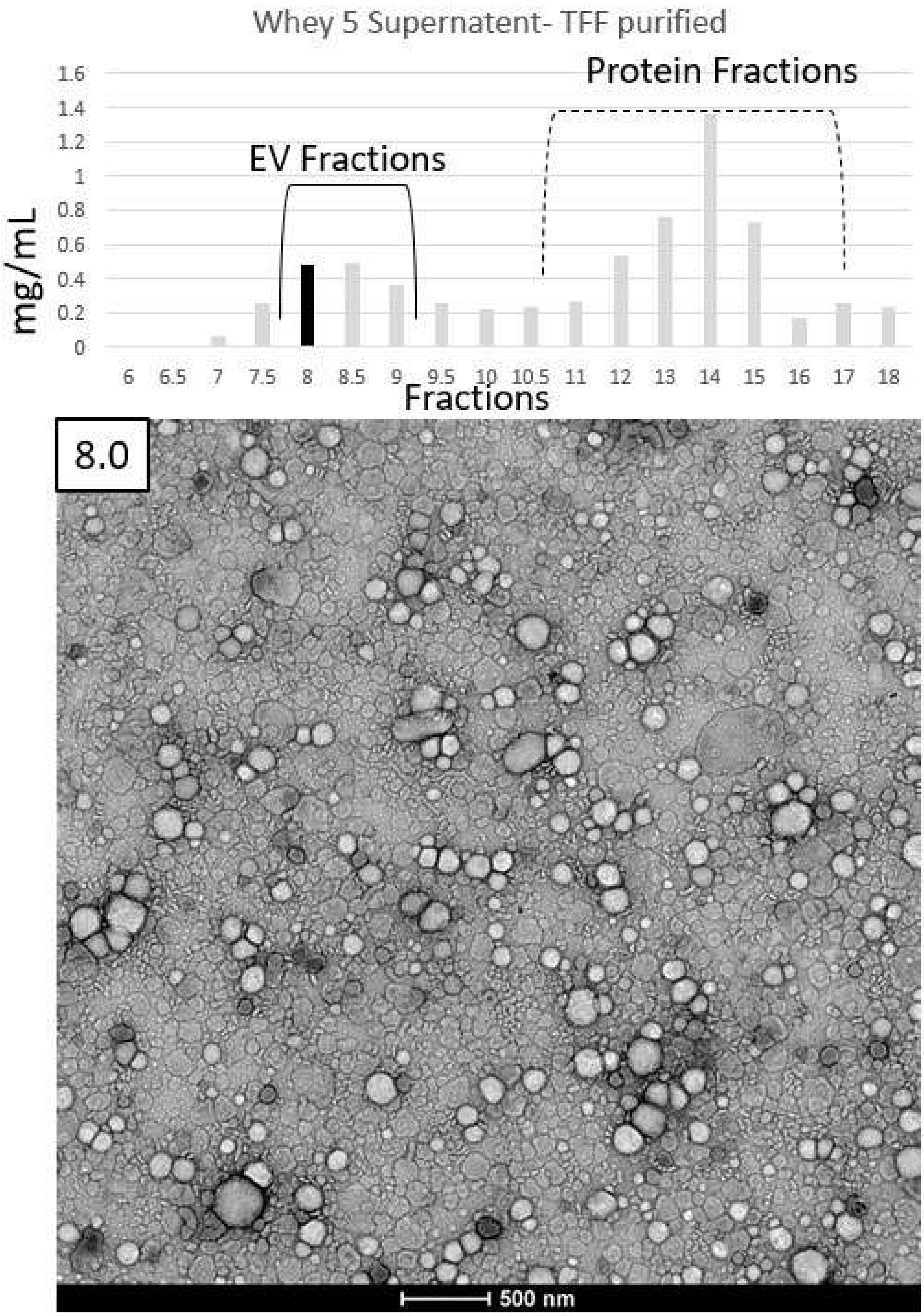

Supplemental Figure 2: Further characterization of potential sources of small EV loss during the UC-based method. The supernatant of the 130,000 RCF spin is discarded in the UC-based method illustrated in Figure 1, with only the pellet from this ultracentrifugation moving forward to EDTA solubilization and SEC separation. We subjected the supernatant of the 130,000 RCF spin to TFF filtration and SEC, and determined that this fraction also displays high numbers of EVs, which again would potentially be lost, not contributing to the final yield of the UC-based method. Top of figure displays sequential fractions collected during SEC filtration step, with protein concentrations on the y-axis in mg/ml. Underneath chart is a negative stain electron microscopy image from fraction 8.0, displaying highly concentrated sEVs left in 130,000 RCF supernatant, reducing efficiency of ultracentrifugation-based procedures.

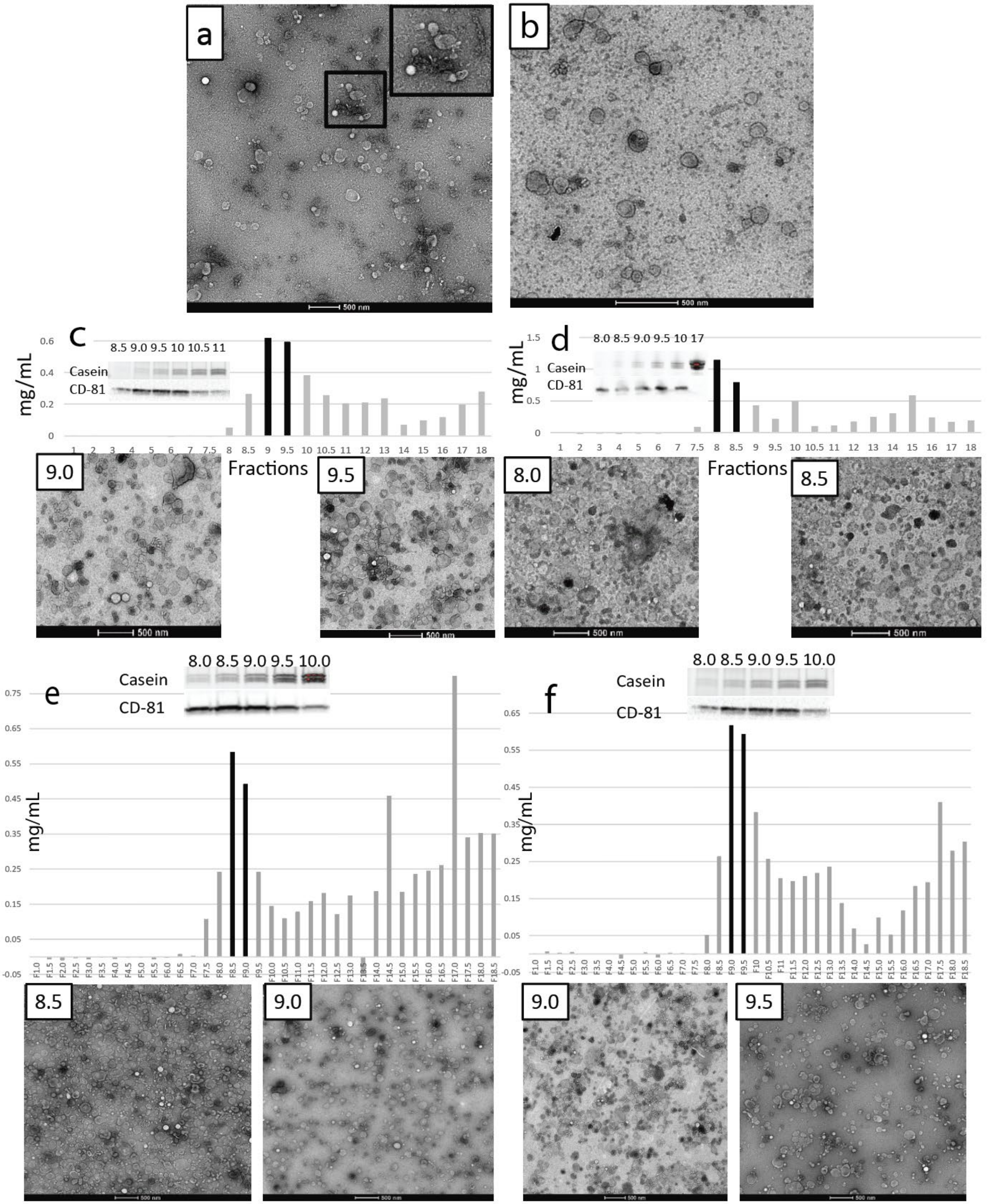

Supplemental Figure 3: Consequences of departures from optimized procedures illustrated in Figures 1 and 2. A) Electron microscopy of ultracentrifugation-isolated sEVs with no EDTA/37°C solubilization step. Note the reduced density of EVs and increased amounts of proteinaceous contaminants, relative to peak EV fractions illustrated in Figures 3 and 4. **B)** Ultracentrifugation-isolated sEVs treated with 30 mM EDTA only for 1 hour, but with no SEC. EVs are relatively sparse

and the preparation is heavily contaminated with protein aggregates. **C)** Electron microscopy of ultracentrifugation-isolated sEVs treated with 30 mM EDTA at room temperature (20°C) for 1 hour prior to SEC separation. CD81 blots and stain-free gels indicate the presence of small EVs, with relatively low levels of Casein contamination. However, by electron microscopy, the EVs in peak SEC fractions are not as dense as seen in the final product of optimal isolations. **D)** Ultracentrifugation isolated sEV's treated at 37°C for 1 hour with no EDTA prior to SEC separation. CD81 blots and stain-free gels indicate the presence of small EVs, but relatively high levels of Casein contamination. Electron microscopy indicates that the EVs are not as dense as in the final product of optimal isolations. **E)** Overview of isolated sEV treated with sub-optimal EDTA levels, i.e. less than 30 mM (shown data used10 mM) results in reduced sEV quantity, purity, and concentration as evidenced by Nanodrop protein quantification and resultant EM of EV fractions 8.5 and 9.0. Gel analysis of fractions for Casein demonstrates the enhanced Casein contamination in sEV fractions, indicating reduced purity, while CD-81 reduction in density and reduced protein concentration suggests a lower yield of sEV's using this reduced EDTA method. **F)** Analysis of reduction in time (shown data used 30 minutes) of EDTA/temperature reaction results in reduced sEV purity, quantity and concentration as evidenced by Nanodrop protein quantification and resultant EM of fractions 9.0 and 9.5. This shift in EV fractions also indicates a slower separation, confirmed by gel-based analysis for Casein. The reduction of time to 30 minutes resulted in decreased yields and delayed sepharose column separation, confirmed by western blot analysis for CD-81 showing low CD-81 prior to fraction 8.5.

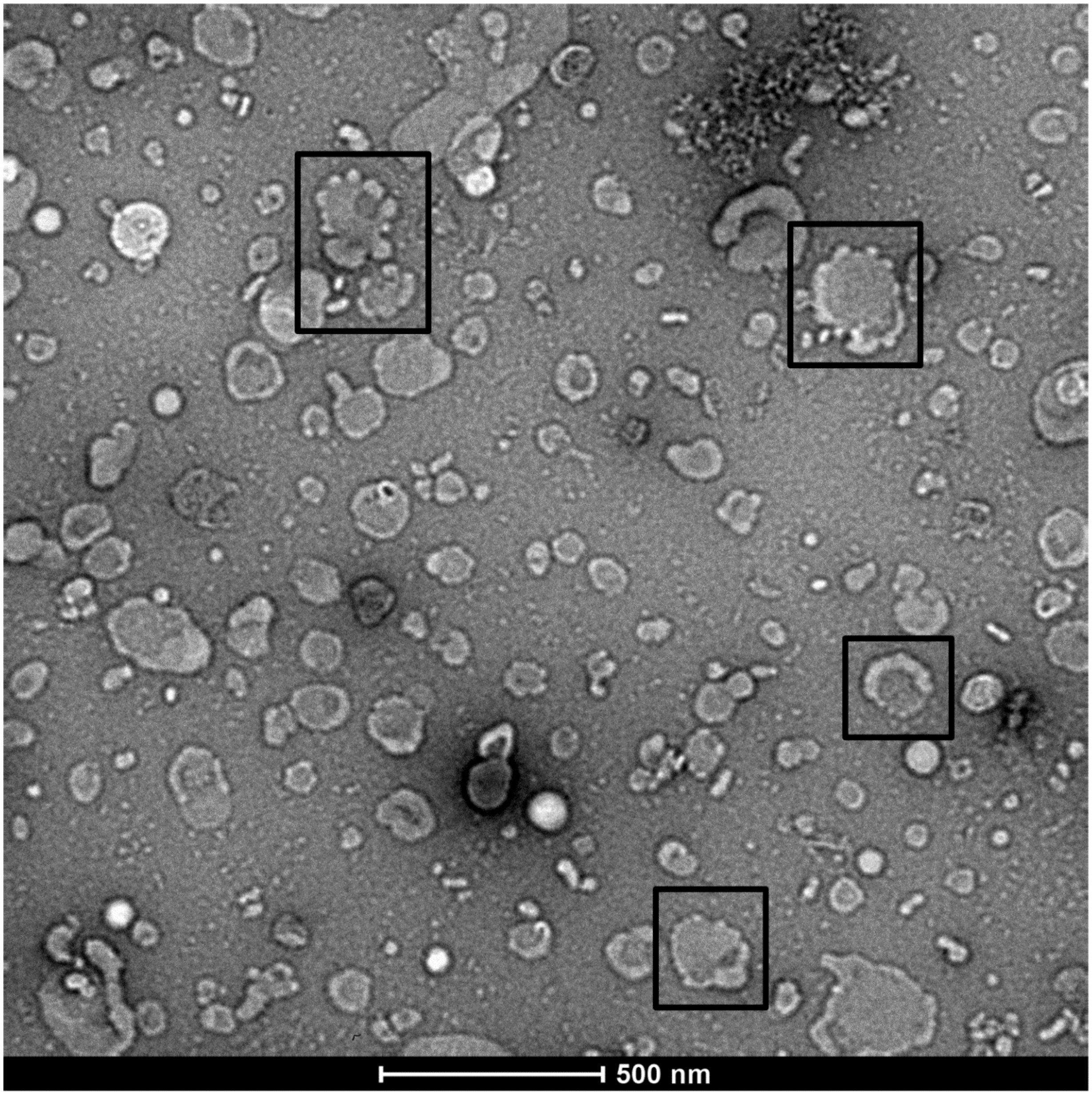

Supplemental Figure 4: High concentrations of EDTA in the solubilization step disrupts EV morphology. Deployment of 100 mM EDTA for 1 hour at 37°C in the solubilization step of a TFF-based protocol appears to cause membrane blebbing and other disruptions to EV membranes.

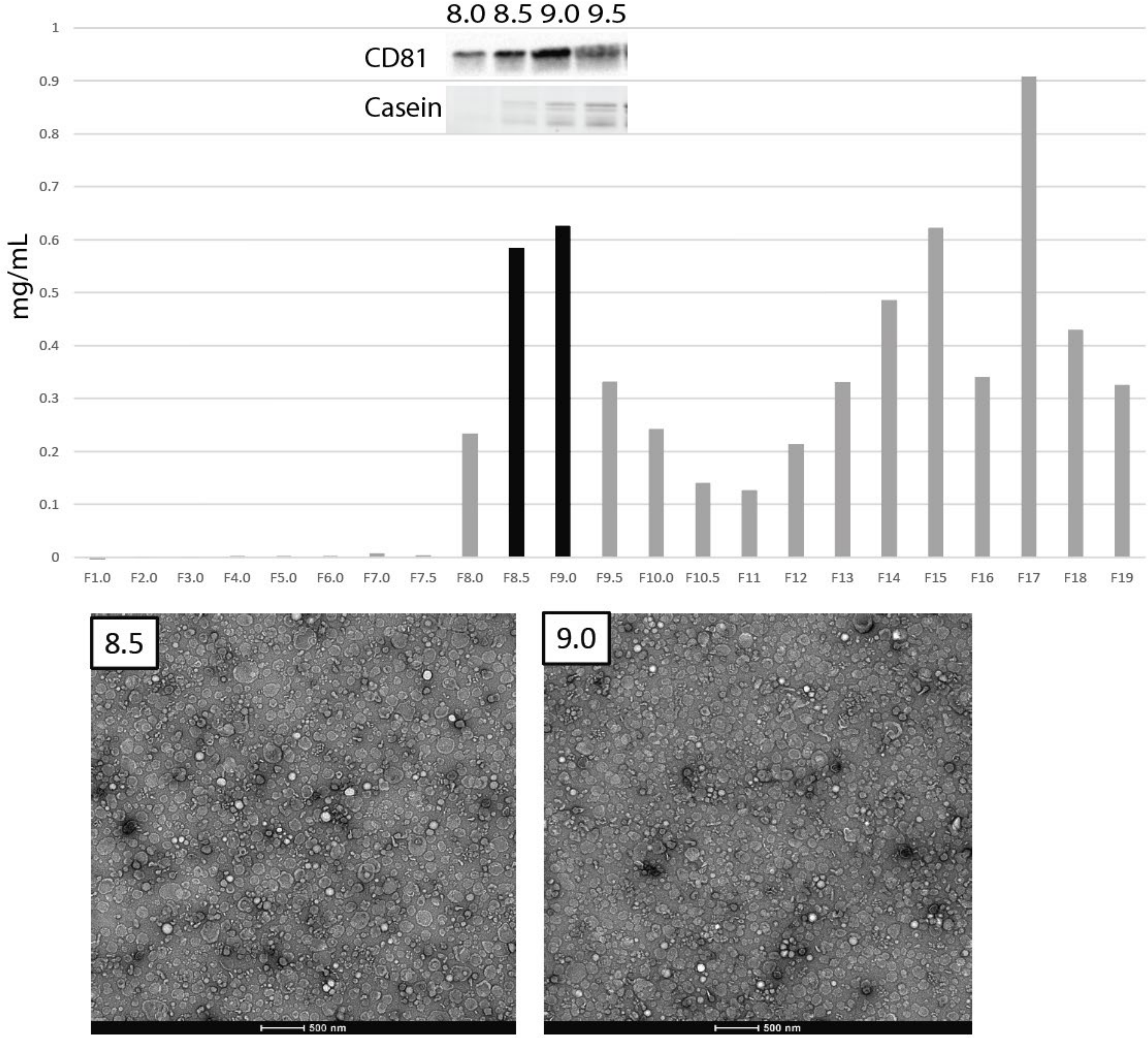

Supplemental Figure 5: **Illustration of the effects of skipping directly to a 130,000 rcf spin after dead-end filtration** (.45 um and .22 um). EV concentrations were reduced by approximately 40%, while Casein contamination was higher than in other UC methods. TEM analysis of EV fractions 8.5 and 9.0 showed higher infiltration of Casein micelles and other proteinaceous contaminants, further highlighting the inadequacy of this method. Additionally, CD81 levels were reduced and shifted to a peak at fraction 9.0, indicating sub-optimal sepharose column separation.
